# A changing pain training landscape: Match trends in NASS-recognized interventional spine and musculoskeletal medicine fellowships versus ACGME/NRMP-accredited pain medicine fellowships (2020–2025)

**DOI:** 10.1016/j.inpm.2026.100765

**Published:** 2026-04-23

**Authors:** Sandy Christiansen, Naileshni Singh, Zachary L. McCormick, Shauna Rakshe, Scott Pritzlaff

**Affiliations:** a Department of Anesthesiology and Perioperative Medicine, Oregon Health & Science University, Portland, OR, USA; b Department of Anesthesiology and Pain Medicine, University of California Davis, Sacramento, CA, USA; c Department of Physical Medicine and Rehabilitation, University of Utah School of Medicine, Salt Lake City, UT, USA; d Knight Cancer Institute Biostatistics Shared Resource, Oregon Health & Science University, Portland, OR, USA

**Keywords:** Pain medicine, Interventional spine, Fellowship, Medical training, Anesthesia, Physical medicine and rehabilitation, Physiatry

## Abstract

**Background:**

In 2023, there was a sudden decline in the popularity of Accreditation Council for Graduate Medical Education (ACGME) Pain Medicine fellowship programs, as demonstrated by National Resident Matching Program (NRMP) application and match data. In comparison, little is known about the application rates and match performance of the North American Spine Society (NASS)-recognized Interventional Spine and Musculoskeletal Medicine (ISMM) fellowships established in 2020.

**Objective:**

We aimed to compare the characteristics of NASS-recognized ISMM applicants and their match results with those of NRMP-sponsored Pain Medicine applicants to determine whether both pathways faced similar challenges in recruiting and successfully matching trainees in recent years.

**Methods:**

This longitudinal observational study examines and describes the total number of applicants, match rate, and position fill percentage results of the ACGME NRMP and NASS ISMM from 2020 to 2025, stratified by gender and primary specialty.

**Results:**

Over the study period, match rates did not markedly change for ISMM, but for ACGME they increased suddenly in 2023 and remained at this level. For ISMM and ACGME, the percentage of positions filled through the match trended downward. For both, the number of female applicants remained low and stable despite fluctuations in the total number of applicants. The distribution of applicants by primary specialties changed for the ACGME; notably, Anesthesiology applicants decreased by 65% from 2021 to 2025, while applications from all other specialties increased, including an increase in Emergency Medicine applicants by 221%. Since its inception, the NASS ISMM match has been primarily composed of Physical Medicine and Rehabilitation (PM&R) physicians, who accounted for 88% of applicants and 90% of successful matches.

**Conclusion:**

ACGME and NASS ISMM pain fellowships have been at the forefront of evolving trends in program fill rates from 2020 to 2025. Match rates for applicants remained high, reflecting a less competitive and more accessible process for those pursuing careers in pain and spine care.

In 2025, male PM&R physicians were the largest group of applicants across both fellowship pathways, highlighting continued strong engagement from this specialty. Although overall applicant numbers have shifted, these trends present an opportunity to broaden outreach, strengthen early mentorship programs, and expand the pipeline of future pain and spine specialists.

## Highlights

• ACGME- accredited Pain Medicine and NASS- sponsored ISMM fellowship programs have experienced shifts in overall fill rates between 2020 and 2025.
• Match rates for both remain relatively high compared with prior years, reflecting a less competitive environment.
• Continued focus on thoughtful recruitment, early mentorship, and sustained engagement will help support workforce stability and ensure that high-quality pain and spine care remain accessible to the patients.

## 1 Introduction

### 1.1 Origins of ACGME and NASS fellowships

#### 1.1.1 Accreditation Council of Graduate Medical Education (ACGME) pain medicine fellowship

In the late 1980s, several primary specialties submitted requests to the American Board of Medical Subspecialties (ABMS) to establish Pain Medicine as an official subspecialty [1]. Despite interest from multiple primary specialties, ABMS ultimately approved the application by the American Board of Anesthesiology (ABA) in 1991. After the approval, the Accreditation Council for Graduate Medical Education (ACGME) established program requirements and accredited the pain fellowship in 1993. In 2013, the Association of Pain Program Directors led the effort to include the Pain Medicine fellowship match in the National Resident Matching Program (NRMP).

Although it is primarily housed within the specialty of Anesthesiology, the Pain Medicine subspecialty naturally benefited from a multidisciplinary approach, reflecting its diverse origins. Consequently, the ABMS expanded the list of primary specialties eligible for board certification in pain medicine to include Neurology (1998), Psychiatry (1998), Physical Medicine and Rehabilitation (PM&R) (2003), Family Medicine (2003), Emergency Medicine (2014), and Radiology (2015) [2]. Only graduates from these residencies are eligible for board certification through ABMS or the ABA.

The NRMP match occurs annually in the fall for matriculation the following summer. It is important to note that the ACGME match involves a two-step certification process. The first step is to apply to individual pain programs through the Electronic Residency Application Service (ERAS), and the second step is to register with the NRMP, which allows applicants to confirm their participation and match with an ACGME-accredited pain program.

#### 1.1.2 North American spine society (NASS) interventional spine and musculoskeletal medicine (ISMM) fellowship

For decades, in addition to ACGME-accredited Pain Medicine fellowships, non-ACGME-accredited fellowships in pain medicine, sports medicine, and spine pain have also trained trainees to treat patients with musculoskeletal and spine pain. The North American Spine Society (NASS), a multidisciplinary organization focused on spine health, sought to unify several high-quality, non-ACGME fellowships to create an official one-year training program in spine and musculoskeletal pain, the Interventional Spine and Musculoskeletal Medicine (ISMM) fellowship.

To achieve this, NASS leadership established baseline metrics for a high-quality program and implemented a formal application process for program admission. The first year of the NASS ISMM match was in 2020. In contrast to ACGME-accredited Pain Medicine Fellowships, NASS ISMM fellowships are primarily housed within the specialty of PM&R. Eligible primary specialties include Anesthesiology, Neurology, Neurosurgery, Orthopedic Surgery, Physical Medicine and Rehabilitation, and Radiology.

The NASS ISMM occurs annually in the summer, for matriculation the following summer. Applicants apply to individual fellowship programs through NASS and then register for the match through NASS, which allows them to confirm their participation and match with a NASS ISMM fellowship program.

### 1.2 Recent changes in ACGME

From 2019 to 2023, matching into an ACGME-accredited Pain Medicine fellowship was consistently considered competitive, until a sudden decline in the specialty's popularity in 2023, as reflected in the 2024 NRMP Match [2].

A suspected cause is the lucrative anesthesiology market, which encourages employment after residency rather than pursuing an additional year of fellowship [2]. ACGME training may not meet the needs of off-cycle applicants and those ineligible for ABA certification, such as internists and those with a surgical background.

### 1.3 Seeking understanding of NASS-recognized ISMM fellowships

Compared with ACGME-accredited Pain Medicine fellowships, little has been published about the match performance of NASS-recognized ISMM fellowships. As a result, it has been unclear whether both the ACGME and NASS pathways face similar challenges in recruiting and successfully matching trainees. Understanding how these two fellowship tracks differ is vital to planning future training capacity.

We aimed to compare the NASS-recognized ISMM match results (2020–2025) with the ACGME NRMP-sponsored Pain Medicine match results (2020–2025). We hypothesized that from 2020 to 2025, NASS would see increases in total applicants, match rates, and fill percentage, whereas the ACGME would see decreases in all three.

## 2 Methods

This longitudinal observational study examines applicant and program characteristics for the ACGME ERAS/NRMP and NASS ISMM matches from 2020 to 2025. We investigated trends in applicant numbers, match rates, program fill percentage, gender representation, and the primary specialty distribution of applicants. Data from AAMC ERAS applicants, the NRMP, and the ISMM Match were obtained from their respective organizations. The Oregon Health & Sciences University Institutional Review Board (IRB) waived oversight of this study (STUDY00028749), as it was determined not to be human subjects research.

The match rate reflects the percentage of applicants who successfully matched into a fellowship program. For ACGME programs, two match rates were calculated each year: the ERAS match rate and the “official” NRMP match rate. The ERAS match rate was calculated as the number of matched applicants divided by the total number of ERAS applicants; the NRMP match rate was calculated as the number of matched applicants divided by the total number of certified NRMP applicants. For the ISMM, the match rate was calculated as the number of matched applicants divided by the total number of applicants.

To compare the match rates directly between NRMP and ISSM, we chose to use the ERAS match rate because it reflects the initial application process, which is more akin to the ISMM application process that does not involve the two-step certification process, described in the introduction. The fill percentage indicates the percentage of positions or programs that were successfully filled through the match process and was calculated by dividing the positions or programs filled by the total offered that year.

Notably, programs in both fellowship pathways fill positions post-match for vacancies, so there are more matriculants within each fellowship pathway than represented by the match data. The ACGME Pain Medicine Match does not have a formal “scramble” or centralized post-match process. After Match Day, the NRMP releases lists of unfilled programs and unmatched applicants, and programs and candidates communicate directly to fill any remaining positions.

In contrast, NASS ISMM program conducts a more structured outreach process after their match cycle concludes. However, precise post-match position fill data are unavailable. As such, the exact number of matriculants into each fellowship pathway falls somewhere between the total number of positions offered and the total number of positions filled by the match, likely closer to the total number of positions offered based on the knowledge and experience of the authors of this study.

Gender was denoted in the application as male, female, or other/unspecified. Other/unspecified applicants included those who did not denote a gender or selected a non-male/non-female answer. Gender match rates were calculated annually by dividing the number of matched male, female, or “other” applicants by the total certified applicants for the NRMP or by the total applicants for the ISSM. Notably, the NRMP began tracking gender match data in 2023, and these data include only individuals in the primary specialty of anesthesiology.

Race and ethnicity data were not analyzed because the ISMM has not historically collected them, and thus they are not available for comparison with the ACGME data.

The data were tabulated in Microsoft® Word (Version 2507), and statistical calculations and figures were generated using R (version 4.5) [3]. The data reported are population data, and the reported proportions represent true population proportions rather than estimates derived from samples; as such, there is no uncertainty and thus no need for 95% confidence intervals. Furthermore, we did not perform statistical testing to compare proportions, as these are the true population proportions rather than estimates derived from samples; no consideration of uncertainty was necessary, and differences were interpreted through a contextual rather than statistical lens.

## 3 Results

### 3.1 Match results (2020–2025)

From 2020 to 2025, the NRMP match included a total of 2915 ERAS applicants. In 2020, there were 514 ERAS applicants; by 2025, this number had decreased to 442, reflecting a 14% decline. ERAS match rates steadily increased from 66% in 2020 to 75% in 2025. Fill percentages decreased from 97% in 2020 to 86% in 2025 (Table 1).

**Table 1:** NASS ISMM and ACGME ERAS/NRMP overall applicant and program match results.

		2020	2021	2022	2023	2024	2025
**ISMM**	ISMM Applicants	74	73	91	82	83	52
	Total Matched	40	45	42	44	43	32
	Positions Offered	43	49	56	56	62	55
	Match Rate^†^	54%	62%	46%	54%	52%	62%
	Positions Filled^Δ^	93%	92%	75%	79%	69%	58%
**ERAS/NRMP**	ERAS Applicants	514	540	548	446	425	442
	NRMP Applicants	395	428	415	359	341	355
	Total Matched	337	362	358	332	320	333
	Positions Offered	349	378	377	393	389	389
	ERAS Match Rate^∞^	66%	67%	65%	74%	75%	75%
	NRMP Match rate^‡^	85%	85%	86%	93%	94%	94%
	Positions Filled^Δ^	97%	96%	95%	85%	82%	86%
	Programs Filled^€^	90%	90%	89%	70%	68%	75%

Legend - †: Matched Applicants/Total Applicants; Δ: Positions Filled/Positions Offered (does not include post-match scramble); ∞: Matched Applicants/Total ERAS Applicants; ‡: Matched Applicants/Total Certified NRMP Applicants; €: Programs Filled/Total Programs. ISMM = Interventional Spine and Musculoskeletal Medicine, ERAS = Electronic Residency Application Service, NRMP = National Resident Matching Program.

From 2020 to 2022, 10%, 10%, and 11% of NRMP programs did not fill, respectively. In 2023, there was a sudden change as 30% of programs went unfilled, leaving 16% positions vacant. In 2024, this trend persisted, with 32% programs remaining unfilled and 18% of positions remaining unmatched. In 2025, 25% programs failed to fill through the match, leaving 14% positions unfilled.

The NASS ISMM match included 455 applicants from 2020 to 2025. The fewest applicants were 52 in 2025, while the most were 91 in 2022. Match rates ranged from a low of 46% in 2022 to a high of 62% in 2021. In 2025, the match rate was 62%. The year with the highest percentage of fellowship positions filled was 2020 at 93%. Since 2020, the percentage of positions filled through the match has steadily declined to 58% in 2025 (Table 1).

### 3.2 Gender distribution (2020–2025)

The number of female ERAS applicants increased from 101 in 2020 to 111 in 2025, an increase of 10%. Male applicants decreased by 20% during the same period, from 413 to 331 (Table 2). Overall, from 2020 to 2025, the gender distribution of applicants was 78% men and 22% women. Male anesthesiologist match rates were 56%, 53%, 54% in 2023, 2024, and 2025, respectively. Corresponding female anesthesiologist match rates were 16%, 14%, and 19%, respectively.

**Table 2:** NASS ISMM and ACGME NRMP applicants and match results by gender.

		2020	2021	2022	2023	2024	2025
**ISMM** Applicants by Gender	Male	56	47	53	55	45	29
	Female	5	14	13	7	8	4
	Not Reported^£^	13	12	25	20	30	19
**ISMM** Matched by Gender	Male	32	31	29	36	27	22
	Female	1	9	7	3	5	0
	Not Reported^£^	7	5	6	5	11	10
**NRMP** Applicant by Gender	Male	413	418	430	351	319	331
	Female	101	121	118	95	106	111
	Other^β^	0	1	0	0	0	0
**NRMP** Matched by Gender (Anesthesiology Only)	Male	N/A^×^	N/A^×^	N/A^×^	204	182	180
	Female	N/A^×^	N/A^×^	N/A^×^	57	48	67

Legend - × : NRMP only began tracking gender match data in 2023 and only within the primary specialty of anesthesiology; £: Gender not selected on application; β: Gender selected as “other” on application.

Overall, from 2020 to 2025, the gender distribution of ISMM applicants was 63% male, 11% female, and 26% unspecified gender. Among those who successfully matched, the distribution was 72% male, 10% female, and 18% unspecified gender (Table 2).

### 3.3 Primary specialty distribution (2020–2025)

From 2020 to 2025, Graph 1 and Table 3 show ERAS anesthesiology applicants decreased from 304 to 106 (−65%); while PM&R physician applicants increased from 124 to 170 (37%). Emergency Medicine applicants increased from 19 to 61 (221%). The number of neurology applicants increased from 11 to 16 (45%). All other primary specialties increased from 55 to 89 applicants (62%).

**Graph 1:**
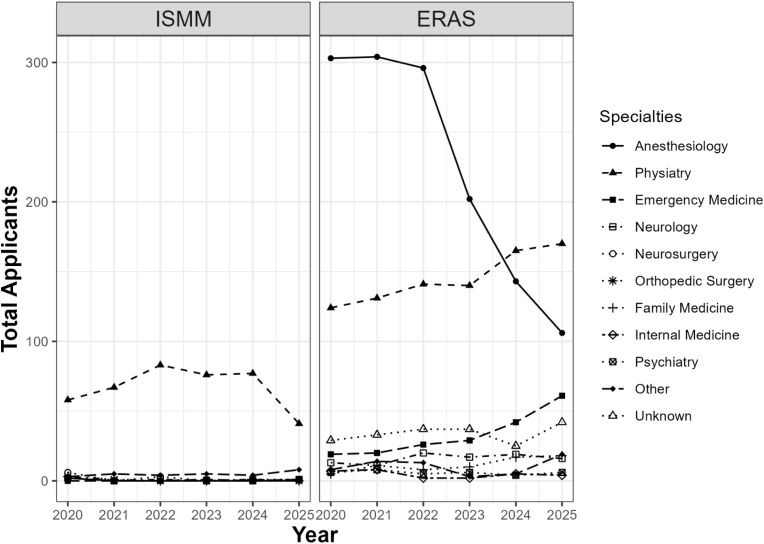
NASS ISMM and ACGME ERAS applicants by primary specialty.

**Table 3:** NASS ISMM and ACGME ERAS/NRMP applicants and match results by primary specialty.

			2020	2021	2022	2023	2024	2025
**ISMM** **Primary Specialty** Matched (*M*) Unmatched (*U*	Physiatry	*M*	39	44	41	41	42	28
		*U*	19	23	42	35	35	13
	Anesthesiology	*M*	0	0	0	0	0	0
		*U*	2	0	0	0	0	0
	Emergency Medicine	*M*	0	0	0	1	0	0
		*U*	0	0	0	0	0	1
	Neurology	*M*	0	0	0	0	0	0
		*U*	2	0	1	0	0	1
	Orthopedic Surgery	*M*	0	0	0	0	0	0
		*U*	3	1	0	0	1	0
	Neurosurgery	*M*	0	0	0	0	0	0
		*U*	6	0	3	0	1	1
	Other	*M*	1	1	1	2	1	4
		*U*	2	4	3	3	3	4
**ERAS** **Primary Specialty**	Physiatry		124	131	141	140	165	170
	Anesthesiology		303	304	296	202	143	106
	Emergency Medicine		19	20	26	29	42	61
	Neurology		13	11	20	17	19	16
	Family Medicine		5	11	8	10	17	18
	Internal Medicine		7	8	2	2	5	4
	Psychiatry		6	8	5	6	4	6
	Other		8	14	13	3	5	19
	Unknown^●^		29	33	37	37	25	42

Legend – *M:* Matched*; U:* Unmatched*;* ●: Applicant did not select primary specialty on application.

The ISMM match is primarily filled by PM&R physicians, who account for 88% of applicants and 90% of successful matches. All other specialties had very few applicants; from 2020 to 2025, there were 2 Anesthesiologists, 2 Emergency Medicine physicians, 4 Neurologists, 5 Orthopedic Surgeons, and 11 Neurosurgeons. Out of these 24 non-PM&R applicants, only 1 Emergency Medicine physician successfully matched (Graph 1 and Table 3).

## 4 Discussion

ACGME and NASS ISMM fellowships are both one-year programs that train physicians to treat patients with chronic musculoskeletal and spine pain. ACGME Pain Medicine differs in that the curricula provide a broad, multidisciplinary education including the biopsychosocial approach to complex pain syndromes, cancer pain management, and inpatient acute pain. In contrast, NASS-recognized ISMM fellowships focus on outpatient musculoskeletal and spine care, prioritizing high-volume interventional procedures, musculoskeletal ultrasound, and electrodiagnostics.

Additionally, the vast majority of ACGME programs remain housed within anesthesiology departments, reflecting the specialty's origins more than 60 years ago and suggesting an enduring structural alignment with anesthesiology-trained physicians.

Given the overlap in the curricula and eligible primary specialties, it is likely that fellowship pathways compete for some applicants, particularly physiatrists, though the extent of the competition is unknown. Based on the knowledge of the authors, graduates from both training pathways pursue and take jobs in all practice settings, including academic hospitals, hospital-based practices, and private practices, and the scope of their practice likely reflects their training.

Although ACGME fellowships experienced a sudden decline in popularity in 2023 [2], little was known about NASS ISMM applicants and match results since the inception of this pathway.

### 4.1 Decreasing volume of applicants

First, both the ISMM match and NRMP were oversubscribed in 2020, with more applicants than available positions; the ISMM had 1.7 applicants per position, and the NRMP had 1.1 applicants per position.

However, since 2023, the ACGME programs have experienced a marked decline in the number of applicants. In 2025, the NASS ISMM program saw a similar drop.

In 2025, for the first time, both programs had more available positions than applicants. For the ISMM match in 2025, 55 positions were available, with only 52 applicants, resulting in a 5% surplus of unfilled positions. For the NRMP match in 2025, there were 389 available positions for only 355 NRMP-certified applicants, creating a 9% surplus.

### 4.2 Match rate and fill percentage

Comparing the “match rate” to the “fill percentage” reveals interesting trends within the NRMP and NASS ISMM matches.

The match rate is affected by the proportion of applicants who successfully complete the process, from submitting the initial application through interviewing to ranking programs. A low match rate can be linked to applicant attrition or program selectivity (i.e., programs choosing not to rank a given applicant) but can also be secondary to an abundance of applicants vying for available positions. The attrition rate is unknown for the NASS ISMM program, but for the ACGME pathway, from ERAS application to NRMP application, attrition remained steady around 20% over the study period.

The fill percentage may further clarify the match's competitiveness by showing the outcomes of the training positions. A low fill percentage may suggest a decline in the specialty's popularity or a growing preference among applicants to find positions outside of the traditional match process; whereas a high fill rate may indicate high competitiveness.

Together, the match rate and fill percentage may indicate the training pathway's popularity, the ability to successfully recruit desired candidates, and whether the growth in “supply” (number of positions) matches the “demand” (number of applicants). Therefore, a high match rate combined with a low fill percentage would suggest a decline in the specialty's popularity through the traditional match process.

Over the six-year period, the match rates for the NASS ISMM fellowship program remained relatively stable around an average of 55%, while the NRMP match rates for ACGME steadily increased with an average of 70%. Meanwhile, the fill percentage of the ISMM match has declined since 2020, from 93% to 58%. Similarly, the NRMP fill percentage has declined from 97% in 2020 to 86% in 2025.

Overall, these trends raise concerns about the declining popularity of both fellowship pathways through the matches. However, what is presently unknown is what percentage of the unfilled positions are filled outside the match processes. Thus, we are ultimately unable to infer whether the decrease in fill percentage is secondary to a decrease in demand in pain training, or rather a decrease in utilization of the formal match process, or both.

### 4.3 Demographic and primary specialty trends

In terms of demographics, both datasets show persistent gender disparities, though we advise caution when interpreting this data, as a large proportion of ISMM applicants listed unspecified gender, and there was limited gender match data available through the NRMP. Over the past six years, females accounted for only 11% of NASS ISMM fellowship applicants and 22% of applicants to ACGME Pain Medicine fellowships. The underrepresentation of women in these fellowships underscores the need for targeted outreach and mentorship; otherwise, gender disparities will weaken the specialty.

The 65% decline in anesthesiology applicants for ACGME Pain Medicine fellowships, along with a 221% increase in emergency medicine applicants, and a 37% rise in physiatry applicants, indicates a shifting composition of applicants and potentially the future workforce. For the first time, in 2024, physiatry had more applicants than anesthesiology by 22 applicants, and this gap widened in 2025 to 64 applicants, a 191% change.

The ISMM fellowship, on the other hand, is almost entirely composed of male physiatrists, with few applicants from other primary specialties and genders, and even fewer successful matches among them.

Comparing the demographic makeup of the applicants, it is likely that ACGME and NASS both compete for male PM&R physician applicants, but not for applicants of other genders or primary specialties. Both pathways would benefit from targeted recruitment of candidates from diverse primary specialties and of women.

### 4.4 Training options outside of ACGME and NASS ISMM

It is important to recognize that there are other training options in pain management, spine care, and musculoskeletal medicine besides ACGME and NASS that may also attract candidates. ACGME Sports Medicine fellowships exist; interventional and neuroradiology programs also offer spine and musculoskeletal fellowships. Lastly, private and hospital-based practices may offer non-accredited one-year training opportunities with various blends of pain, spine, musculoskeletal, and sports medicine.

There are also certification courses available through various pain societies, some of which are industry-sponsored. These courses provide exposure to advanced interventions, including neuromodulation and minimally invasive spine procedures. It will be critical to identify and track these additional training pathways to gain a comprehensive understanding of the state of pain, spine, and musculoskeletal medicine training in the United States.

## 5 Conclusion

The NRMP and NASS ISMM applicant and match results indicate an increase in unfilled programs and positions, although many of these positions were likely filled through their respective post-match “scramble” processes. Both pathways exhibit gender disparities and PM&R as the dominant specialty.

Customized strategies are needed to ensure an adequate future workforce in Pain Medicine, such as expanding pain education across multiple specialties and improving recruitment, mentorship, and diversity efforts. This will be essential to maintain a strong and diverse workforce for the aging and medically complex population living with chronic pain.

## Disclosures

Dr. Christiansen serves on the Board of Directors for the Pacific Spine and Pain Society.

Dr. Singh receives royalties from Wolters Kluwer.

Dr. McCormick serves on the Board of Directors of the International Pain and Spine Intervention Society (IPSIS), has research grants from Avanos Medical, Boston Scientific, Presidio Medical, Saol Therapeutics, Spine Biopharma, SPR Therapeutics, Stratus Medical (paid directly to the University of Utah), and previous consultancies with Avanos Medical, Saol Therapeutics, Stryker, and OrthoSon (all relationships ended).

Dr. Rakshe has no conflicts of interest to disclose.

Dr. Pritzlaff is a paid consultant for SPR Therapeutics, Medtronic, Bioventus, Sharp Biomedical, and Wise Neuro; receives royalties from Wolters Kluwer and Oxford University Press. He receives educational grants from Medtronic, Nevro, Abbott, and Biotronik (paid directly to the University of California, Davis). He is a co-founder of FlywheelRx.

## Funding

The authors have no sources of funding to declare for this manuscript.
